# Supplementary material for: Angelica sinensis polysaccharide as potential protectants against recurrent spontaneous abortion: focus on autophagy regulation
Source: Front Med (Lausanne). 2025 Jan 15;12:1522503. doi: 10.3389/fmed.2025.1522503 (PMC11774876; doi:10.3389/fmed.2025.1522503)
Supplement: Supplementary material S1 — The main instruments used during the LC-MS process, along with their models/specifications and manufacturers. [file Supplementary_file_1.zip › Metabolomics sequencing data FC1.2/8.代谢通路分析/Exposure_Ctrl/up/气泡图-top20.pdf]

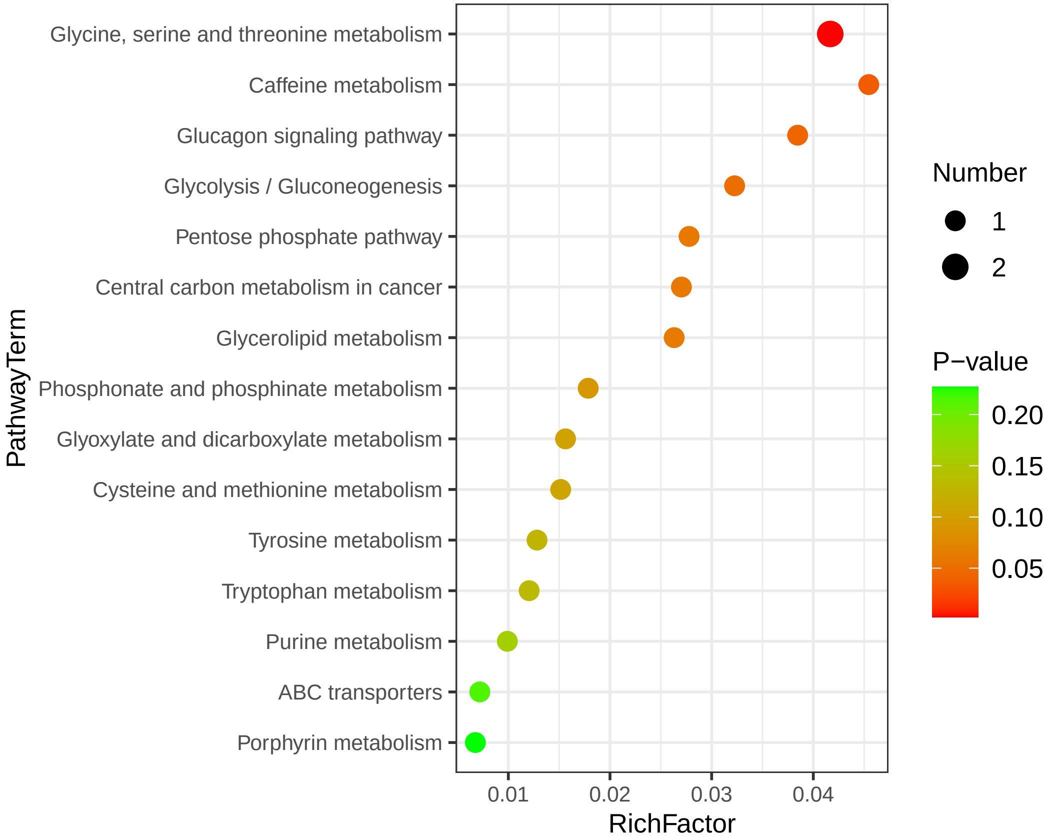

Glycine, serine and threonine metabolism
Caffeine metabolism
Glucagon signaling pathway
Glycolysis / Gluconeogenesis
Pentose phosphate pathway
Central carbon metabolism in cancer
Glycerolipid metabolism
Phosphonate and phosphinate metabolism
Glyoxylate and dicarboxylate metabolism
Cysteine and methionine metabolism
Tyrosine metabolism
Tryptophan metabolism
Purine metabolism
ABC transporters
Porphyrin metabolism
PathwayTerm
0.01
0.02
0.03
0.04
RichFactor
Number
1
2
P−value
0.20
0.15
0.10
0.05
